# Editorial: Big data and machine learning in sociology

**DOI:** 10.3389/fsoc.2023.1173155

**Published:** 2023-05-09

**Authors:** Heinz Leitgöb, Dimitri Prandner, Tobias Wolbring

**Affiliations:** ^1^Institute of Sociology, Leipzig University, Leipzig, Germany; ^2^Institute of Sociology, University of Frankfurt, Frankfurt, Germany; ^3^Institute of Sociology, University of Linz, Linz, Austria; ^4^Institute of Labour Market and Socioeconomics, University of Erlangen-Nuremberg, Nuremberg, Germany

**Keywords:** big data, machine learning, computational social science, digitalization, artificial intelligence, social science research methodology

## Introduction

The dawn of the digital age, aptly characterized by “computers everywhere” (Salganik, 2018, p. 3), has shaped modern societies and, thus, the lives of individuals worldwide in unique ways. The ubiquity of the internet, in conjunction with the mass distribution of a variety of affordable internet-enabled digital devices, has created new possibilities for collecting, storing, linking, sharing, and exchanging information. Also, the massive progress in computer performance regarding processing capacities and computational speed has paved the way for advances in programming which culminated in the recent progress in artificial intelligence (AI) research, referred to as the recent AI spring (for a brief outline of the history of AI research, see, e.g., Mitchell, 2019). Its results are—among others—the deep-learning-induced successes in speech and object recognition that enable processes as complex as simultaneous translation or autonomous driving. The societal consequences range from the emergence of new professions, business fields, leisure activities, behavioral cultures, and associated lifestyles to new social inequalities (digital divide), dependencies (digital and data literacy gaining relevance as key competencies), and forms of deviant/criminal activity (e.g., cyberbullying and -crime, online hate speech, crimes organized/executed through the internet).

This digital revolution affects the social sciences in various ways. First, social processes experience fundamental change and adaption that require extensive scientific elaboration. Second, the steadily increasing application of digital technologies generates an enormous mass of finely granulated data in various forms and formats. It is not just that enormous amounts of data can now be easily accessed and analyzed. Digital innovations have allowed the collection of data in various formats that were previously difficult to compile (e.g., georeferenced data, tracking or process data, intensive longitudinal data, social media text data; Golder and Macy, 2014; Leitgöb and Wolbring, 2021). This digitization and datafication of society have shaped empirical social science research fundamentally in recent years and will continue to do so. Third, the increasing computational power and the maturation of software environments have promoted the development of algorithmic solutions for complex statistical problems. It paved the way for the nascent field of computational social science (CSS; e.g., Lazer et al., 2009, 2020; Edelmann et al., 2020; Engel et al., 2022a,b) at the intersection of the social sciences, statistics, informatics, and mathematics.

The future viability of the empirical social sciences will largely depend on their ability to adapt to the conditions associated with the ongoing digitization of society (Wolbring, 2020). While new digital technologies have provided empirical social research with unique opportunities for data generation and analytical processing, they also impose new methodological challenges that shape research designs, theoretical foundations, and the methods used. For example, using digital process data for scientific purposes requires the development of tailored data and measurement theories, quality criteria, and corresponding quality assurance procedures to establish quality standards comparable to those from survey methodology. Also, this shift in perspectives afflicts the way the obtained data are typically analyzed, raising the question of how to transfer the relevant advancements from computer science to social science methodology (Törnberg and Uitermark, 2021; Jarvis et al., 2022).

Against this backdrop, the Research Topic covers two core elements of CSS, (i) *big data* and (ii) *machine learning*. While this editorial focuses on the big picture, highlighting some key aspects in both areas without purporting to represent a comprehensive review, the research papers published in this Research Topic provide detailed insights into the unfolded content area. We organize the remaining part of the editorial according to the three perspectives typically addressed in the discussion of the impact of digitalization on social science research: the epistemological perspective (Section 2), the data perspective (Section 3), and the data analytical perspective (Section 4).

## Epistemological consequences of digitalization

There are multiple competing epistemological concepts in the discussions about CSS (e.g., Törnberg and Uitermark, 2021). While the relevance of data and the potential consequences of “big data” for the social sciences were first addressed long before societal digitization, it was the digitalization wave of the late 20th century that brought the discussion to a broader part of the scientific communities. At the beginning of the millennium, both social scientists and statisticians stated that it is necessary to discuss the impact which computer science had on the emerging CSS and reflect on the consequences of analyzing social phenomena through a “computational paradigm of society” (Törnberg and Törnberg, 2018).

As a naïve starting point, it can be assumed that digital data and their traces are *true* and, thus, exact representations of social processes. As such, digital data would be naturally emerging data representing the real underlying structure of society and social interactions. This view mirrors how computer scientists not necessarily capture but often handle digital trace data in practice: Pursuing a data and performance-driven research agenda, they focus primarily on the algorithmic optimization of predictions by specifying models that are superior to others concerning predictive accuracy but with few concerns regarding the included measures (e.g., by considering selection effects and measurement error). While trying to trace the complex networks and data flows that shape modern societies and economies in much greater detail and to establish causal inferences beyond traditional methods, they tend to be less preoccupied with the data generating process, including aspects of research design or protocol (Allen et al., 2017).

In contrast, a more realistic view would neglect the idea of natural data. As Lazer et al. (2014, p. 1203) highlighted: “quantity of data does not mean that one can ignore foundational issues of measurement and construct validity and reliability and dependencies among data”. All digital platforms are designed by humans within certain societal constraints to measure and often even monetize social interactions, resulting in structures that potentially manipulate individuals (Mayer-Schönberger and Cukier, 2013; van Dijck, 2014; Couldry and Mejias, 2021). Research has shown that empirical studies can disadvantage minorities or groups of low social status unless they adhere to a strict definition of fairness and justice (e.g., Mitchell et al., 2021) and theoretical reasoning (Mullainathan and Spiess, 2017; Molina and Garip, 2019). Accordingly, big data and AI-driven research need to be embedded into theoretical frameworks and enable transparent discussions about how data are biased. Algorithms can also be sensitive to contextually problematic conceptualizations and depend on interactional settings. This can be highly impactful for the generation and reproduction of social inequalities as “one of the core competencies—and responsibilities—of the social sciences” (Gerdon et al., 2022, p. 2; see also Section 4).

Nevertheless, scholars pursuing these ideas certainly see much benefit in the increased amount of available data, the rich granularity, and new types of measures. Likewise, they are eager to integrate new data sources and methods into their theoretical work, but they will interpret their results more carefully and reflected and deal critically with the limitations of their data. Developing and expanding a social scientific perspective (e.g., Blei and Smyth, 2017) on the implementation of big data and AI-driven analysis into the research processes is an essential complement to the more technical focus of disciplines such as informatics and mathematics, which sociology and related social science disciplines can contribute to the fields of CSS and data science. In the context of this Research Topic, such issues are also at the forefront of several articles examining how good or fair automated classification and decision-making processes can be. The studies of Kuppler et al. (in this volume) and Seewann et al. (in this volume) examined how new methods and techniques could support social scientific work but also expressed their concerns about ethics and limits attached to such methods.

## Digitalization and the big data era

The datafication of society is a consequence of the digital revolution. In contemporary societies, individuals leave digital traces in numerous processes, such as communication, mobility, shopping, banking, dating, working, and learning (Lazer et al., 2009; for a review see Golder and Macy, 2014). These digital behavioral data (DBD) increase at an exponential rate (Jarvis et al., 2022, p. 35). Typically, they are collected and processed by institutions such as public administration, non-governmental organizations, and commercial companies. They differ in some relevant respects from scientifically produced data in quantitative social research, such as survey data and experimental data.

First, they differ in size. DBD are available in incredible quantity, allegorized as “data deluge”. Second, DBD are omnipresent, often generated continuously and available in real time. Third, DBD do not represent some homogeneous data type, but differ considerably in form, format and complexity (e.g., dimensionality and structuredness). Their diversity ranges from social media text and respective metadata (Hadler et al.; Schünemann et al.; Schwitter et al. in this volume), social network and interaction data, data from webpages (Seewann et al. in this volume), online consumer behavior data, geocoding (Nguyen et al. in this volume) and time references, physical condition and mobility data, internet search engines results, to information extracted from images and videos. Accordingly, DBD fall under the minimal definition of “big data,” typically characterized by the three Vs: (i) huge in *volume*, (ii) high in *velocity*, and (iii) diverse in *variety* (Laney, 2001; Beyer and Laney, 2012).

The systematic use of DBD and other digitalized mass data (e.g., contextual data from ecological systems, large-scale digitalized register, administrative and official statistical data) for scientific purposes marks the beginning of a big data era (e.g., Kitchin, 2014; Connelly et al., 2016) in the social sciences. Many advantages of this development are obvious (for overviews, see, e.g., Golder and Macy, 2014; Adams and Brueckner, 2015; Cesare et al., 2018). Foremost, a tremendous amount of data containing fine-grained and often high-dimensional information about social phenomena at different societal levels, which are impossible to collect with traditional non-digital procedures, is potentially accessible now. What once was a rare commodity in science is now ubiquitous (Golder and Macy, 2014; Salganik, 2018) and is often systematically stored in massive social data archives. However, Connelly et al. (2016, p. 1) argue that it is “not the size or quantity of these data that is revolutionary. The revolution centers on the increased availability of new types of data which have not previously been available for social science research”. This allows under-addressed research questions to be answered. And the systematic linkage of DBD, also with various other data sources (e.g., survey, register, official statistics and contextual data, e.g., Christen et al., 2020; Klumpe et al., 2020; Stier et al., 2020), entails additional analytical boost. For example, see the contributions of Hadler et al., Nguyen et al., and Schünemann et al., in this volume. Furthermore, DBD are expected to be less prone to errors induced by reactivity because they are often collected unobtrusively in the background without social interaction with others (e.g., Harari et al., 2017; Salganik, 2018; Diekmann, 2020; Keusch et al., 2022).

However, the scientific use of DBD is also associated with various challenges. DBD are typically produced for administrative, commercial, or other purposes outside the academic field or as the by-product of everyday digital processes. Thus, DBD do not necessarily meet scientific quality standards (Salganik, 2018), and their application in a research context presupposes the critical evaluation of—among others—conceptual fit (Do the observed variables adequately map the theoretical constructs of interest?), measurement quality, and representation to avoid bias that invalidates the conclusions. However, while well-established (missing) data and measurement theories, error models, and relevant quality criteria are readily available for scientific data, this is usually not the case for DBD. The first important contributions to this topic were provided by Hsieh and Murphy (2017), Amaya et al. (2020), Biemer and Amaya (2021), and Sen et al. (2021). Furthermore, rigorous inferences from empirical data greatly benefit from systematically implemented research designs that determine the data-generating process (e.g., Wolbring, 2020). For example, causal effects cannot simply be learned from a joint distribution of observed variables (Pearl, 2010). It also requires theoretical elaboration and a research design that rules out threats to internal validity, such as confounding, endogeneity, and systematic selection. In other words, “design trumps analysis” (Rubin, 2008, p. 808) in causal effect identification. However, the generative process of DBD does not, in principle, rely on such design considerations, limiting their usability for the causal inference task and frequently resulting in very noisy data (e.g., Silver, 2012).

Finally, it is also worth noting that progress in portable digital and sensor technologies offers unique opportunities in academic research to collect DBD about individuals' everyday practices and routines. App-based survey tools allow for the active and passive collection of DBD and their systematic combination with online survey data (e.g., Jäckle et al., 2019; Keusch et al., 2019; Kreuter et al., 2020). For participant recruiting, non-probability samples particularly online access panels are expected to play a decisive role and require extensive investigation (e.g., Cornesse et al., 2020).

## The turn in data analysis

Opportunities to collect and use data of previously unknown mass, granularity, and complexity, in new formats and based on non-scientific and unknown data-generating processes require analytical models that adequately address these data characteristics (e.g., Amaturo and Aragona, 2019; Edelmann et al., 2020). In recent years, impressive computer hardware innovations regarding storage capacities, computing power, interconnectedness, task division, and data transmission evoked the development of such computationally intensive statistical software solutions, creating an algorithmic culture of statistical modeling without assuming an underlying stochastic data model as in the traditional statistical modeling culture (Breiman, 2001). This algorithmic culture is strongly affected by machine learning, a field of sub-symbolic AI research dominated by informatics but with substantive roots in statistics (Friedrich et al., 2022).

Machine learning (ML) lacks a precise definition, being “as much a culture defined by a distinct set of values and tools as it is a set of algorithms” (Grimmer et al., 2021, p. 397). Besides processing numerical data, ML algorithms are also developed to process text data. This is demonstrated by some articles in this volume (Haensch et al.; Munnes et al.; Egger and Yu). For a comprehensive overview of the various ML algorithms, see the textbooks of Bishop (2006), Hastie et al. (2009), Goodfellow et al. (2016), Mohri et al. (2018), Sutton and Barto (2018), Jurafsky and Martin (2023), Murphy (2022).

The field is broadly classified into two domains: supervised and unsupervised learning. Although both share the automated extraction of information from data, they differ in their learning objectives. Supervised ML utilizes labeled output data *Y* and input data *X* to learn the input-output mapping for predictive and regression purposes. In contrast, the primary purpose of unsupervised ML is to detect and describe systematic patterns (latent structures) in input data *X* without labeled output data *Y*. However, this binary classification of ML approaches is neither disjoint nor exhaustive (Molina and Garip, 2019). While some ML algorithms can be used in both domains, others belong to neither. The latter is—among others—the case for reinforcement learning and some speech and language processing algorithms. Furthermore, some algorithms can be principally assigned to one domain, but contain features from the other. An example is generative adversarial networks (GANs), classified as unsupervised ML models because no human labeling of the input data is required. However, GANs are trained on the principle of self-supervision; that is, the algorithm initiates a data labeling process to solve some classification problems. A typical field of application for GANs is manipulating audio or video material producing deepfakes (Eberl et al. in this volume). It is also worth noting that many algorithms subsumed under the ML paradigm already have a long social science research tradition but are not explicitly designated as an ML application. Prominent examples are linear modeling, hierarchical agglomerative and *k*-means clustering, *k*-nearest neighbor algorithms, principal component analysis, and neural network analysis.

As outlined, the primary goal of supervised ML applications is the prediction of Ŷ from *X*. In contrast, the traditional stochastic statistical modeling approach, referred to as “generative modeling” (Donoho, 2017), focuses on parameter estimation. That is, on the generation of $\hat{\beta }$, which represent the estimated effect sizes of the effect of *X* on *Y* (Mullainathan and Spiess, 2017). It requires specifying the functional form of the joint distribution of *X* and *Y* (Athey and Imbens, 2019). This modeling perspective is in line with the epistemic focus on causal explanation, particularly with the tasks of causal inference and generative mechanism detection (e.g., Gangl, 2010; Hedström and Ylikoski, 2010; Imai et al., 2011; Winship and Morgan, 2015). It leads to “simple and interpretable models” (Molina and Garip, 2019, p. 29) that mimic the data-generating process. These models are based on strict theoretical assumptions, tied to a set of testable propositions (Grimmer et al., 2021). However, ML-based prediction models are much more data hungry (e.g., the simulation study of van der Ploeg et al., 2014) and complex, with up to millions of parameters and more opaque input-output-functions (Grimmer et al., 2021) that “produce black-box results that offer little insight on the mechanism linking the inputs to the output” (Molina and Garip, 2019, p. 29). The primary objective is predictive accuracy maximization in out-of-sample (training data) conditions, provoking data-driven *ad hoc* modeling decisions without substantial theoretical foundation (Radford and Joseph, 2020). This has relevant implications for the applicability of ML algorithms in sociology.

(i) For explanatory purposes, ML modeling strategies require conceptual and technical optimization to generate valid interpretable results that illuminate the generative social mechanisms based on massive amounts of DBD (e.g., the discussion in Radford and Joseph, 2020; Hofman et al., 2021; Breznau, 2022). This includes an adequate construct-measurement match and measurement modeling (Jacobs and Wallach, 2021).

(ii) The data deluge and the availability of data-driven ML algorithms for analytical processing evoked a *debate on the relevance of (social) theory*. The positions range from “the end of theory” and “correlation supersedes causation” proclamations (e.g., Anderson, 2008) to the call for a strong emphasis on theoretical reasoning to counteract technical limitations, problematic assumptions, limited interpretability, and false conclusions (e.g., Radford and Joseph, 2020; Wolbring, 2020). In any case, prominent examples such as the mispredictions of Google Flu Trends (e.g., Butler, 2013; Olson et al., 2013; Lazer et al., 2014) illustrated the demand for a flexible methodological framework with theory, traditional data sources and methods, as well as DBD and algorithmic approaches as complementary elements to be integrated to maximize knowledge gain (Lazer et al., 2014; Schnell, 2019). Also, unsupervised ML algorithms as exploratory tools could contribute to the inductive process of theory development.

(iii) ML algorithms optimized for prediction offer an opportunity to extend the key epistemological goals in sociology. While the prediction task has so far only played a minor role alongside the explanation task (e.g., Chen et al., 2021), its relevance has become particularly evident during the COVID-19 pandemic (e.g., Pavlović et al., 2022). The pandemic situation required predicting the consequences of strict policy measures (e.g., social distancing, the closing of schools, lockdowns) on various aspects of social life (e.g., student learning outcomes, mental health issues, domestic violence, social and economic inequalities, poverty) to support policy decision making (e.g., Jahn et al., 2022). In addition, Watts (2014) argued that the development of theory and causal explanations could also benefit from a stronger focus on prediction in sociology.

(iv) Assessing the quality of (out-of-sample) predictions requires respective performance metrics. Alongside the traditional technical measures (e.g., accuracy, precision, sensitivity, specificity, AUC, e.g., Steyerberg, 2010), increasing importance is attached to “social” metrics. These account for predictive fairness by quantifying the total amount of bias (for a typology of potential biases at the intersections between data, algorithms, and users, see Mehrabi et al., 2021) that causes a diverging predictive performance across and statistical discrimination against specific groups along ascriptive attributes, such as gender, age, and ethnicity. Although several fairness criteria have been developed based on different definitions of fairness (for an overview, see, e.g., Caton and Haas, 2020; Mitchell et al., 2021; Han et al., 2022; Pessach and Shmueli, 2022), additional concepts with respective evaluation criteria are needed to assess the overall social impact of algorithmic predictions on decision-making in detail. Sociology can play a decisive role in developing such a conceptual framework (e.g., Gerdon et al., 2022; Starke et al., 2022). An example is provided by Kuppler et al. (in this volume), advocating a conceptual differentiation between algorithmic fairness and distributive justice.

## Outlook

This editorial highlights the digital revolution's impact on social sciences—particularly on empirical sociology—from an epistemological, data, and analytical perspective. In line with the thematic orientation of the Research Topic, it focuses on big data and machine learning, which are two core elements of the nascent and interdisciplinary field of computational social science (CSS). Building on Lazer et al. (2020) and Leitgöb and Wolbring (2021), we finally share some thoughts on the institutional processes required to establish this computational turn as a sustainable success story.

(i) *Universities need to adopt their institutional structures and facilities to meet the demands*. This includes an organizational restructuring to facilitate interdisciplinary collaboration and the financing of the computational infrastructure mandatory for the storing, linking, and high-speed processing of massive amounts of data under the highest security standards.

(ii) *Social science education needs to be reformed*. In particular, the traditional training in methods and methodology, focusing on survey data and classical frequentist statistics must be supplemented by CSS elements based on mathematics, computational statistics, informatics, and data science to maximize the students' (digital) data literacy. Besides training in gathering, processing, analyzing, and visualizing big digital data with software packages such as R or Python, this also includes conducting simulation studies (e.g., Keuschnigg et al., 2018). The success of implementing these topics in the sociology curricula will determine the future viability of the discipline and the extent to which sociology will play a leading role in CSS.

(iii) *Big centralized data infrastructure needs to be established*. This infrastructure is intended to serve the systematic comprehensive collection, processing, and secure storage of any social science data in accordance with legal data protection standards. The main objective is to provide this data to the scientific community for secondary data analysis. In addition to the financial resources, technical innovations, and know-how, this requires a new culture of willing data and code sharing from the stakeholders such as researchers, universities, public authorities, and social media companies (Lazer et al., 2020).

(iv) *Detailed data protection regulations and ethical guidelines are necessary to establish handling security for researchers*. The progressive digital technologies enable researchers to explore, in principle, entirely new methodological pathways in studying social phenomena and generating empirical evidence for decision-making. However, relevant legal and ethical questions still need to be resolved to legitimize the use of these methodological innovations, especially because many data are sensitive or difficult to anonymize (e.g., Salganik, 2018). While legal data protection frameworks are set in principle in most countries (e.g., by the General Data Protection Regulation, applicable in all European Union member states since 2018), there has been uncertainty about how existing legislation will be handled in practice (for some brief examples, see Leitgöb and Wolbring, 2021). Likewise, a comprehensive set of tailored and broadly accepted ethical standards is still unavailable in this developing field of research (e.g., Hand, 2018; Piano, 2020).

(v) *The application of AI innovations in teaching and throughout the research process needs to be regulated*. Current developments, particularly the distribution of the chatbot software ChatGPT (Generative Pre-trained Transformer),^1^ illustrate that AI systems can be used not only for data analysis in the social sciences. Instead, these systems allow a wide range of tasks to be solved throughout the research process and can also be used by lecturers and students in academic training courses. Initial reactions to these innovations range from banning of the use of ChatGPT for students (e.g., at Sciences Po Paris^2^) to active considerations of how to collaborate with generative AI to delegate tasks and maximize knowledge acquisition. In any case, standards should be developed on how to regulate the use of AI systems and how their contribution to scientific work should to be disclosed.

The above aspects outline the key efforts required to provide the CSS agenda with a solid foundation for long-term success. The Research Topic aims to serve as a platform for different contributions to the core elements of CSS: big data and machine learning. Ideally, the Research Topic and its articles encourage further research and contribute to the progress that the digital revolution has brought to social science research methodology.

## Author contributions

All authors listed have made a substantial, direct, and intellectual contribution to the work and approved it for publication.

## Footnotes

^1^For details, see https://openai.com/blog/chatgpt (02/08/2023).

^2^For details, see https://newsroom.sciencespo.fr/sciences-po-bans-the-use-of-chatgpt (02/08/2023).
